# A Microfluidic Device for Tobacco Ringspot Virus Detection by Electrochemical Impedance Spectroscopy

**DOI:** 10.3390/mi14061118

**Published:** 2023-05-26

**Authors:** Xiaoxing Weng, Chen Li, Changqing Chen, Gang Wang, Chenghao Xia, Lianyou Zheng

**Affiliations:** 1 Zhejiang Academy of Agricultural Machinery, Jinhua 321000, China; 2 College of Quality and Safety Engineering, China Jiliang University, Hangzhou 310018, China; 3 Zhejiang Jiu Qi Machinery Co., Ltd., Jinhua 321000, China

**Keywords:** tobacco ringspot virus, microfluidic impedance method, impedance sensor, virus detection

## Abstract

Aiming at the problem of how to achieve the rapid detection of pathogenic microorganisms, this paper takes tobacco ringspot virus as the detection object, designs the impedance detection and analysis platform of tobacco ringspot virus based on microfluidic impedance method, establishes an equivalent circuit model to analyze the experimental results, and determines the optimal detection frequency of tobacco ringspot virus detection. Based on this frequency, an impedance–concentration regression model was established for the detection of tobacco ringspot virus in a tobacco ringspot virus detection device. Based on this model, a tobacco ringspot virus detection device was designed by using an AD5933 impedance detection chip. A comprehensive test study was carried out on the developed tobacco ringspot virus detection device through various testing methods, which verified the feasibility of the tobacco ringspot virus detection device and provided technical support for the field detection of pathogenic microorganisms.

## 1. Introduction

In recent years, the global epidemic caused by the novel coronavirus (COVID-19) virus has drawn wide attention from society to biosafety incidents caused by pathogenic microorganisms and accelerated the speed of research on the detection of pathogenic microorganisms. For the analysis of pathogenic microorganism detection technology, each kind of detection technology has its own advantages and disadvantages: in immunological detection technology [1], the detection process is fast and simple, but the detection accuracy is low; in molecular biology detection technology [2], there is high detection sensitivity and accuracy, but the detection process is complex, and there are high requirements for professional technology; as the latest detection technology, metabolic detection technology [3] has the characteristics of high accuracy, high sensitivity, and simple operation. The purpose of this study is to develop a fast, accurate, and portable pathogenic microorganism detection device. The microfluidic technology [4,5,6] in the pathogenic microorganism metabolism detection technology is taken as the detection principle of the detection device, so as to ensure the detection accuracy and sensitivity, while also having the ability of rapid detection.

The concept of microfluidic technology appeared in 1992, which refers to a system science and technology that uses microchannel at the micron level to detect or operate trace samples to be detected. A microfluidic device, also known as microfluidic chip, refers to the integration of sample preparation, reaction, separation, detection, analysis, and other operations into a chip to automatically complete the whole process of analysis and detection, so as to achieve various functions of conventional chemical or biological laboratories. As the channels in microfluidic chips are microchannel of micron level, the flow state of the fluid in the microchannel channel is mainly affected by the surface tension of the fluid. Thus, the fluid in the microchannel channel has a stable laminar flow state, which will contribute to the stable biochemical reaction in the microchannel [7,8,9].

In this study, tobacco ringspot virus (TRSV), a common plant virus, was selected as the object of microfluidic impedance detection. TRSV is a representative species of Nepovirus of the genus Comoviridae, which belongs to the second class of imported quarantine pests published in China [10]. TRSV has a large number of parasitic hosts, which can infect more than 300 plants in 54 families such as tobacco, soybean, and potato [11,12,13]. Tobacco ringspot virus has a variety of transmission modes [14], a wide range of hosts, and is distributed in nearly 40 countries in the five continents of the world. Therefore, the spread of the virus is harmful and will cause significant economic losses. At present, the main detection methods for identification of the virus are RT-PCR detection and serum detection. In terms of serological relationship, although most TRSV isolates were consistent, there were at least four strains with different serological relationships; in addition, there was a certain serological correlation between TRSV and potato black ringspot virus in this subgroup. The IC-RT-PCR technique combined the characteristics of serum detection and RT-PCR, but it still had potential hidden dangers of misdetection and cross-reaction in serology, which had a certain impact on the accurate detection of TRSV.

Therefore, the purpose of this study is to develop a portable device for the detection of tobacco ringspot virus based on microfluidic impedance method, which can be applied to the rapid and accurate detection of tobacco ringspot virus in the field. At the same time, based on this device, it lays a good foundation for the subsequent detection of other pathogenic microorganisms and provides technical support for the detection of pathogenic microorganisms.

## 2. Materials and Methods

### 2.1. Principle of Microfluidic Impedance Detection

Microfluidic impedance detection technology is based on a microfluidic chip system, which converts the biological virus concentration into electrochemical impedance signals for rapid detection. The detection principle is based on the microfluidic chip system as the platform. Firstly, biological virus antibody molecules are fixed on the surface of the conductive transducer (gold fork array microelectrode) in the microfluidic chip, and then the biological virus antigen solution is injected into the microchannel at the mesoscale. The viral antigen and antibody react in the microchannel to form a specific complex and attach to the conductive transducer. The impedance characteristics of the conductive transducer are changed. Therefore, a quantitative relationship between the change in impedance signal and the concentration of detected substance is established to achieve the purpose of detection [15].

Electrochemical impedance spectroscopy (EIS) was used to analyze the impedance changes in electrochemical electrodes on microfluidic chips [16,17,18,19]. Electrochemical impedance spectroscopy is an important method in electrochemical studies. The electrochemical impedance spectroscopy technique was to apply a small amplitude sinusoidal alternating excitation voltage disturbance signal to the target, measure the ratio of the alternating current (AC) voltage to current signal changes with the sine wave frequency, and obtain the impedance spectrum of the target [20].

The impedance object of this detection was a gold fork finger electrode with a surface binding tobacco ringspot virus antibody and an antigen-specific complex. Detection by electrochemical impedance spectroscopy had the following two technical advantages:

1. The applied perturbation signal was a small amplitude AC voltage. On the one hand, the effect of perturbation on the properties of electrode and viral antigen antibody could be reduced. On the other hand, the relationship between the properties of the electrode surface and the impedance signal was approximately linear, which was convenient for the processing of detection data.
2. Electrochemical impedance spectroscopy had a wide frequency range. Thus, more electrochemical information of electrode interface could be obtained during detection.

### 2.2. Microfluidic Impedance Detection Chips and Systems

**Figure 1:**
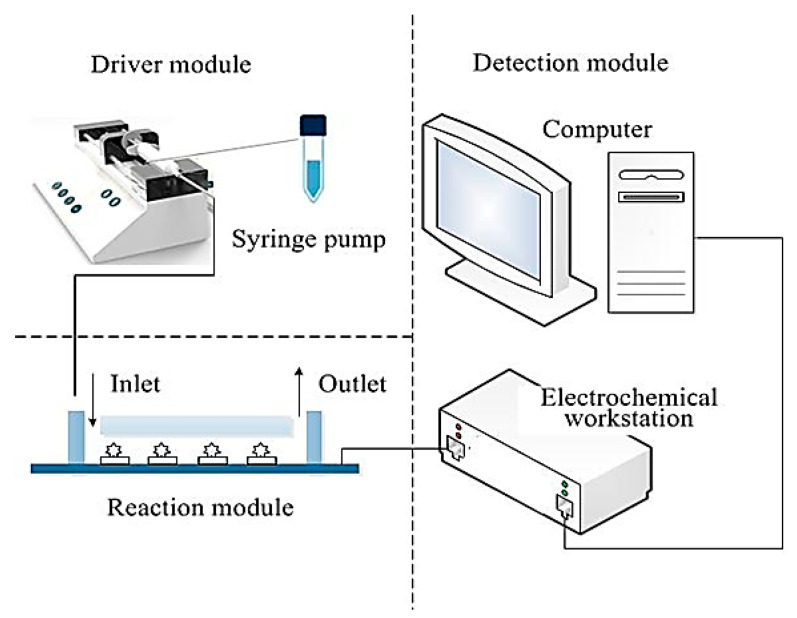
Schematic diagram of microfluidic impedance detection experiment platform.

According to the principle of microfluidic impedance detection and biosensor technology, a tobacco ringspot virus impedance detection system, composed of a driving module, reaction module, and detection and analysis module, was built. The driving module was the microinjection pump (Model Harvard, Harvard Apparatus, Holliston, MA, USA), the reaction module was microfluidic chip, and the detection and analysis module was composed of CS350 electrochemical workstation (Model CS350, Wuhan Coster Technology Co., Ltd., Wuhan, China) and computer. The detection system could realize reagent injection, biological reaction, impedance detection, and analysis functions, as shown in Figure 1.

The parameters of the test instrument were set as follows: (1) The injection speed of the injection pump was 10 μL/min; (2) The excitation voltage of electrochemical workstation was 100 mV, and the test frequency was 1~100 kHz. In total, 10 data points were set within each test frequency order of magnitude, and a total of 50 data points were set. The design dimension parameters of each component of the microfluidic chip are shown in Table 1. The main reagents used in increasing the experiment are shown in Table 2. The core function of the tobacco ringspot virus detection device developed in this study was to detect the impedance signal of the microfluidic chip electrode after the specific reaction between virus and antibody occurred in the microfluidic chip microreaction chamber. The power supply range of AD5933 (Analog Devices, Inc., Wilmington, MA, USA) was 2.7 V~5 V, which provided the basis for the selection of power supply. The normal operating temperature ranged from −40 °C to 125 °C, meeting the temperature requirements in field detection scenarios. CS350 was tested, and the results were obtained. Then, AD5933 was used for the test, and the results obtained at this time were similar to those before. It can be inferred that the application of AD5933 was feasible. Compared with the traditional impedance measurement method, the AD5933 impedance measurement chip integrated the impedance detection function into a single chip, which was easy to operate and had higher accuracy. At the same time, it could also help to reduce the size of the device.

## 3. Results

### 3.1. Impedance Value and Phase Angle Analysis

According to the experimental data obtained from the impedance detection experiment, 50 test points were selected for each test experiment within the set detection frequency range to detect the impedance value and phase, and the following impedance–frequency and phase–frequency relationship diagrams were obtained.

**Figure 2:**
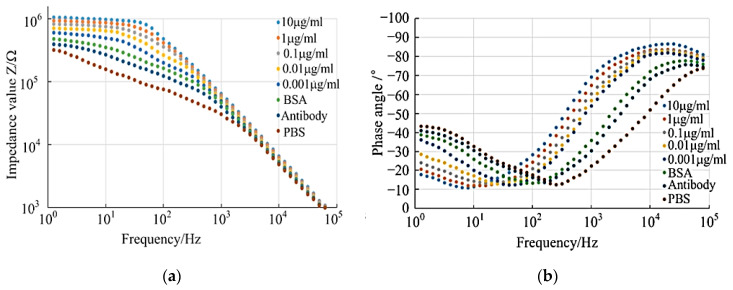
Impedance–frequency and phase angle–frequency curves of different solutions. (**a**) Impedance–frequency. (**b**) Angle–frequency.

According to the analysis of the impedance–frequency diagram (see Figure 2a), the impedance change trend in all test groups decreased with the increase in detection frequency. The overall change trend had a certain similarity, and the overall impedance change trend could be roughly divided into two sections: when the detection frequency was in the frequency band of 1~10^3^ Hz, the change in the experimental impedance values of each group showed a downward trend, and the impedance values of each group had evident differences. When the detection frequency was in the band of 10^3^~10^5^ Hz, the change in experimental impedance values of each group showed a linear trend in decline, and the impedance values of each group were coincident. According to the above image characteristics, it could be seen that the impedance difference of each group of experiments in the 1~10^3^ Hz detection band was the key point of theoretical research and analysis. Combined with the electrical impedance change characteristics, the following analysis was made: it could be seen from Figure 2a that the impedance values of each group increased successively from the bare electrode test group to the 10 μg/mL test group, which proved the antibody sedimentation fixation from the side, blocking the treatment, and the specific reactions of the antibody antigens in the experiment were successful. This was because the obstructing effect on the electrode reaction gradually increased with the increase in the attachment on the electrode’s surface, and, with the progress of the experimental procedure, the attachment on the electrode surface continued to increase. At the same time, the increase in the virus concentration would further promote this effect, which was exactly proved by the differences in the impedance values of each group of experiments in the low-frequency detection band. This analysis needs to be further verified by subsequent equivalent circuit analysis.

From the analysis of the phase–frequency diagram (see Figure 2b), the change in phase value of each test group presented a trend in first decreasing and then increasing with the increase in detection frequency. The overall trend was similar to some extent. When the detection frequency was in the frequency band of 1~10^2^ Hz, the experimental phase of each group showed a downward trend. When the detection frequency was in the band of 10^2^~10^5^ Hz, the experimental phase of each group showed a linear upward trend. According to the electrochemical impedance spectroscopy mechanism, when the phase value was −90°, the detected system was a pure capacitance system. When the phase value was 0°, the detected system was a pure resistance system. According to the phase trend shown in the phase–frequency diagram, in the frequency band of 10^2^~10^5^ Hz, the highest phase value was close to −90°, indicating that the impedance in this frequency band was mainly affected by the capacitance characteristics. The minimum phase angle was close to −10° in the 1~10^2^ Hz frequency band, indicating that the impedance in this frequency band was mainly affected by the resistance characteristics. In addition to the change trend in phase value, it could also be found from Figure 2b that the phase angle front would shift towards the low-frequency region with the increase in tobacco ringspot virus concentration. This change in trend indicated that the influence of resistance on impedance was dominant with the increase in tobacco ringspot virus concentration.

### 3.2. Equivalent Circuit Analysis

#### 3.2.1. Establishment of Equivalent Circuit

**Figure 3:**
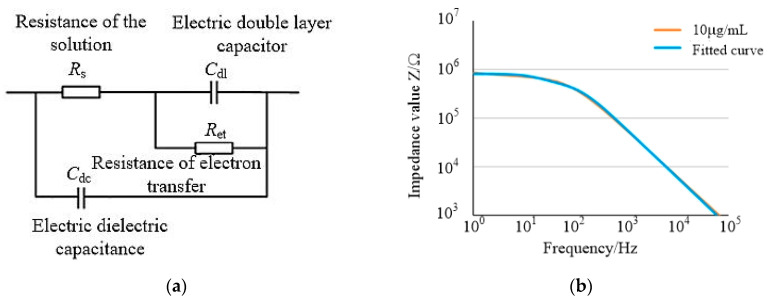
Equivalent circuit and simulation result. (**a**) Diagram of equivalent circuit. (**b**) Fitted curves of impedance and phase angle of 10 μg/mL TRSV.

The obtained electrochemical impedance spectra were analyzed by equivalent circuit analysis, and the equivalent circuit model was designed in Figure 3a. The model of equivalent circuit consisted of solution resistance (*R*_s_), electron transfer resistance (*R*_et_), double layer capacitance (*C*_dl_), and dielectric capacitance (*C*_dc_). The double-layer capacitance *C*_dl_ represented the capacitance of the liquid phase contact surface caused by ions in a layer of charge formed on the upper surface of the electrode when the detection reagent contacted the electrode. Electron transfer resistance *R*_et_ represented the resistance generated when the electron transfer occurred on the surface of the electrode. The parallel connection between the double-layer capacitor *C*_dl_ and the electron transfer resistance *R*_et_ represented the impedance generated between the electrode surface and the solution. *R*_s_ represented the resistance of the test reagent; *C*_dc_ was the capacitance of the electrochemical cell.

The equivalent circuit model constructed by Zview software was fitted by complex nonlinear least square method [21]. Taking the TRSV virus impedance of 10 μg/mL as an example, Figure 3b shows the TRSV virus impedance data curve of 10 μg/mL and the impedance curve fitted to the equivalent circuit. By comparing the two data curves, the average error of impedance value was 0.7% and the maximum error was 11.3%. The mean phase angle error was 0.8%, and the maximum error was 5.3%. In the 10^−3^ μg/mL to 1 μg/mL group, the average impedance errors were 0.7%, 1.3%, 1.1%, 0.6%, respectively. The average phase angle errors were 1.4°, 0.9°, 0.5°, 0.8°. The low error of each experimental group proved that the equivalent circuit model had a good fitting consistency with the impedance detection system constructed in the experiment.

When the detection frequency was in low-frequency band and high frequency band, respectively, the electrode impedance presented two different impedance characteristics, which corresponded to the electrical characteristics of the two branches in the equivalent circuit model. When the detection frequency was in the low-frequency band, the voltage frequency in the low-frequency band could not make the specific complex conduct, which was regarded as insulation. In the equivalent circuit, the current did not pass through the branch of the dielectric capacitor *C*_dc_, and the impedance at this time was determined by the branch of *R*_s_ + *R*_et_ + *C*_dl_. When the detection frequency was in the high frequency band, the voltage frequency in the high frequency band could make the specific complex conduct electricity. In the equivalent circuit, the current passed through the branch of the dielectric capacitor *C*_dc_. In this case, the impedance was controlled by the *C*_dc_ branch.

#### 3.2.2. Effect Analysis of Equivalent Circuit Element on Impedance Value

Under the experimental parameters of each group, the equivalent circuit model was simulated via complex non-linear least squares method to further study the mechanism of electrode impedance change. The bare electrode impedance test data were used as the control group in the simulation test, and the simulation data of each equivalent circuit component were shown in Table 3.

It can be seen from Table 1 that the dielectric capacitance *C*_dc_ of the solution changed little, by only −3.74% compared with the control test, when the TRSV virus concentration increased to 10 μg/mL. Solution resistance *R*_s_ increased from 201.32 kΩ to 560.15 kΩ, with a range of 178.23%. Electron transfer resistance *R*_et_ increased from 67.57 kΩ to 253.52 kΩ, with a range of 275.20%. The double-layer capacitance *C*_dl_ decreased from 196.07 nF to 34.42 nF, with a variation of −82.45%.

At high band rate, the impedance was mainly the *C*_dc_ of the solution dielectric capacitance, which remained unchanged, indicating that the impedance decreased with the increase in frequency, and there was no significant difference in the impedance values of viruses at different concentrations. At low frequencies, when the virus concentration increased, the *C*_dl_ of the double-layer capacitor decreased significantly, and the average impedance value was about 43.5 kΩ, accounting for about 7.6% of the total impedance value, indicating that the change in the *C*_dl_ of the double-layer capacitor had no significant effect on the impedance change.

The results showed that the solution resistance *R*_s_ and the electron transfer resistance *R*_et_ increased with the increase in virus concentration, accounting for 94.6% of the total impedance changes, indicating that solution resistance *R*_s_ and electron transfer resistance *R*_et_ had important effects on the impedance changes. The increases in solution resistance *R*_s_ and electron transfer resistance *R*_et_ were due to the combination of the tobacco ringspot virus antibody with the tobacco ringspot virus, which made part of the electrode surface of the microfluidic chip not in contact with the solution, which impeded the electron transfer between the electrodes and the movement of the ions between the electrodes.

In conclusion, it was feasible to detect the concentration of tobacco ringspot virus by detecting the change in the impedance value of the microfluidic chip electrode, and it had a good detection effect under the conditions of low-frequency detection.

### 3.3. Detection of TRSV Concentration

Better detection frequency conditions were helpful to obtain better detection results of virus concentration, so as to improve the detection accuracy of the developed tobacco ringspot virus detection device. In order to determine the optimal detection frequency, the impedance–frequency diagram obtained could be analyzed, and the impedance difference between each concentration gradient was used as the selection criteria. The larger the impedance differences between different concentration gradients the better the detection effect of the tobacco ringspot virus at this frequency.

**Figure 4:**
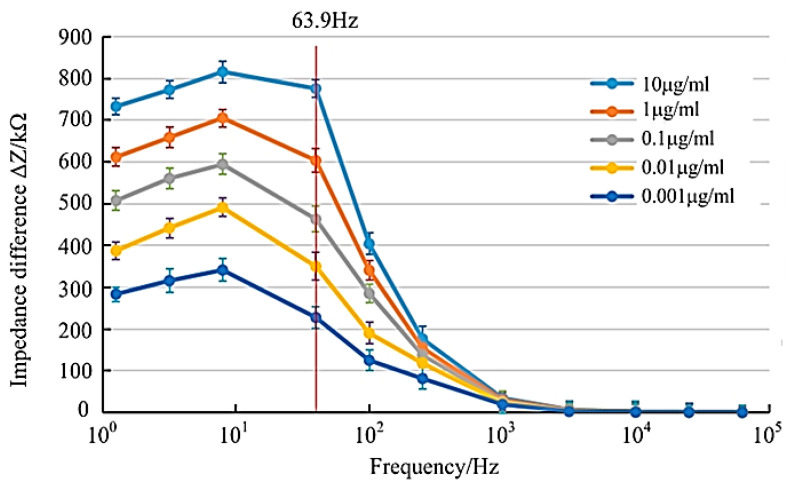
Mean impedance difference of virus concentration.

It can be seen from Figure 4 that the average impedance difference changed as follows: on the whole, it first increased and then decreased; in the low-frequency band, the average impedance difference increased first and then decreased. When the frequency reached 63.9 Hz, the average impedance difference between each concentration group had the largest difference, and the frequency was used as the optimal excitation voltage frequency of the tobacco ringspot virus detection device.

**Figure 5:**
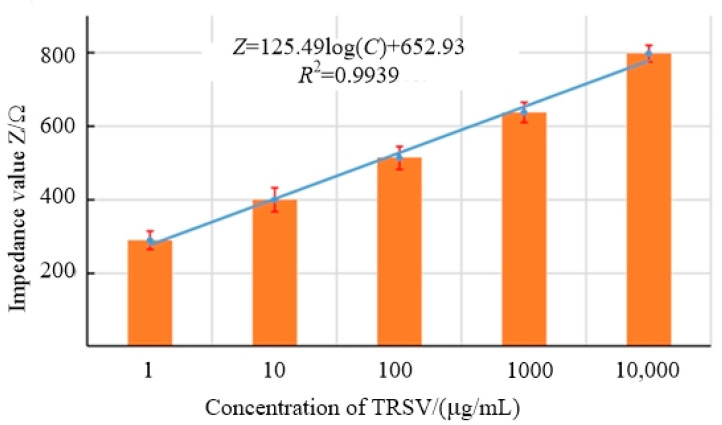
Concentration–impedance at the optimal detection frequency.

After determining the optimal detection frequency, the impedance–concentration regression model of the tobacco ringspot virus detection device should be established under this frequency. The excitation voltage of the electrochemical workstation was set as 63.9 Hz, and impedance detection experiments were conducted on tobacco ringspot virus of each concentration group. Impedance values were obtained within the virus range of 0.001 μg/mL~10 μg/mL at the optimal detection frequency, as shown in Figure 5.

Through software fitting, the regression detection model was established based on virus concentration *C* and impedance value *Z*, and the detection limit was 0.57 μg/mL.

(1) $$Z=125.49\log\left(C\right)+652.93\;\;\;\begin{array}{c} \left(R^{2}=0.9939\right) \end{array}$$

### 3.4. Detection Device Test

After the hardware assembly and software debugging of the detection device, the actual detection performance should be tested, i.e., the stability, reliability, and specificity of the detection results of the device, and the detection effect should be compared with other traditional detection effects.

#### 3.4.1. Stability Test

Under the same experimental conditions, the stability of the tobacco ringspot virus detection device was verified by detecting the impedance values of three groups of fixed resistors. From the analysis of the test results of the stability of the three groups (see Table 4), the impedance detection values of each resistor under the detection frequency of the three groups were stable and did not fluctuate significantly, and the measured impedance values were accurate, which could prove that the detection stability of the tobacco ringspot virus detection device was good.

#### 3.4.2. Reliability Test

**Figure 6:**
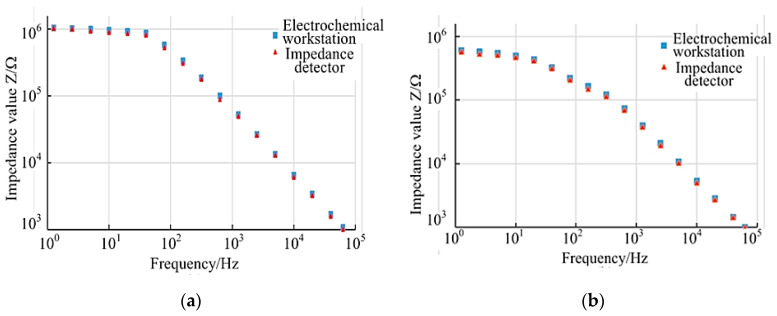
Comparison of impedance values of the two detection devices at different concentrations. (**a**) at 10 μg/mL (**b**) at 0.001 μg/mL.

Under the same experimental conditions, the electrical impedance of the tobacco ringspot virus detection device was compared with that of the electrochemical workstation to verify the reliability of the tobacco ringspot virus detection device. According to the comparative experimental results of the detection of tobacco ringspot virus in the two groups (see Figure 6), the impedance values of the tobacco ringspot virus detected by the tobacco ringspot virus detection device and the impedance values of tobacco ringspot virus detected by the electrochemical workstation were basically consistent, which proved that the tobacco ringspot virus detection device had good reliability.

#### 3.4.3. Specificity Test

Under the same experimental conditions, the impedance values of the different plant viruses and virus mixed solutions were detected by the tobacco ringspot virus detection device for comparison. Meanwhile, the reliability of the tobacco ringspot virus detection device was verified by combining the display feedback of the detection device.

**Figure 7:**
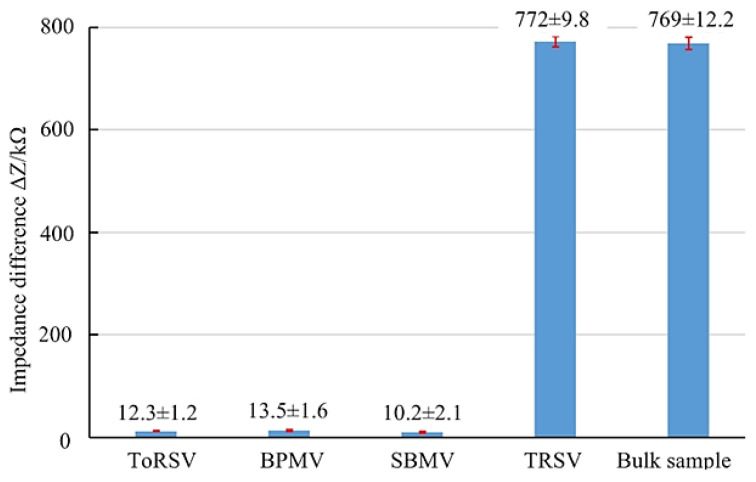
Variation in impedance of different viruses.

It can be seen from Figure 7 that the impedance difference of the virus mixed sample (Tomato ringspot virus, ToRSV; Bean pod mottle virus, BPMV; Southern bean mosaic virus, SBMV) was basically consistent with that of the tobacco ringspot virus solution, while the impedance of the virus solution of the other three groups was close to that of the negative control group, and the display result of the detection device was also consistent with the analysis result of the impedance difference. From the above analysis, it could be seen that the tobacco ringspot virus detection device developed by this research could accurately detect the tobacco ringspot virus in the samples. This result proved that tobacco ringspot virus had good specificity for detecting the tobacco ringspot virus.

Table 5 shows the comparison results between the tobacco ringspot virus detection device and other traditional detection technologies. The detection time of the tobacco ringspot virus detection device is shorter when the chip pretreatment is completed. It can be seen from the comparison that the tobacco ringspot virus detection device has the characteristics of a low detection limit, short detection time, and strong detection specificity.

## 4. Conclusions

1. In view of the complicated operation of virus detection, the correlation between the concentration of tobacco ringspot virus and the impedance of tobacco ringspot virus at a certain detection frequency was found, and the impedance–concentration regression formula of tobacco ringspot virus was established, which solved the problem of the complicated and time-consuming processing of impedance data in the measurement of virus concentration.
2. Aiming at the difficulty in rapid detection of pathogenic microorganisms in the field, a tobacco ringspot virus detection device based on microfluidic impedance biosensor technology was developed. The device had the advantages of a low detection limit, short detection time, and strong detection specificity, and solved the problem of a lack of rapid detection means in the field detection of pathogenic microorganisms.
3. In the performance test of tobacco ringspot virus, the detection device was used to detect three sets of fixed resistance values of 100 kΩ, 500 kΩ, and 1 MΩ, and stable and correct impedance values were obtained, which proved that the tobacco ringspot virus detection device had good stability. In the control experiment between the detection device and the electrochemical workstation to detect TRSV virus with five groups of step concentrations, the experimental impedance results showed good consistency, which verified that the tobacco ringspot virus detection device had good reliability. The impedance detection experiments of different virus solutions and mixed virus solutions were carried out with the detection device. The experimental results showed that the TRSV virus solutions and mixed TRSV virus solutions could cause specific changes in chip impedance values, while other virus solutions did not, which verified that the tobacco ringspot virus detection device had good detection specificity. Compared with other traditional detection techniques, the tobacco ringspot virus detection device showed the advantages of a low detection limit, short detection time, and strong detection specificity. The tobacco ringspot virus detection device had good detection performance and could provide technical support for the detection of pathogenic microorganisms and guarantee for biosafety.

## Figures and Tables

**Table 1:** Design dimension parameters of each component of microfluidic chip.

Microfluidic Chip Components	Dimension Parameter
Microchannel	30 mm long, 0.3 mm wide, 100 μm deep
Storage tank and waste tank	Diameter 3 mm, 100 μm deep
Gold fork finger electrode	The number of electrode pairs was 20, the length of electrode was 0.6 mm, the width was 15 μm, the height was 100 nm, and the spacing of each electrode was 15 μm
Microreaction chamber	1.2 mm long, 0.3 mm wide, 0.1 mm high
External electrode plate	5 mm long, 5 mm wide

**Table 2:** List of main reagents used in the experiment.

Reagent Name	Specification
Tobacco ringspot virus leaves	500 mg
Monoclonal antibody to tobacco ringspot virus	0.05 mg/mL
Bull Serum Albumin solution (BSA solution)	1%
phosphate-buffered solution (PBS)	0.1 mol/L, PH 7.4

**Table 3:** Analog data table of each electronic component of equivalent circuit.

TRSV Cons (μg/mL)	*R*_s_ (kΩ)	*R*_et_ (kΩ)	*C*_dl_ (nF)	*C*_dc_ (nF)
Control	201 ± 2	68 ± 0.6	196 ± 2	2.9 ± 0.02
0.001	204 ± 2	78 ± 0.7	144 ± 1	2.9 ± 0.02
0.01	220 ± 2	161 ± 2	102 ± 1	3.0 ± 0.03
0.1	279 ± 3	188 ± 2	63 ± 0.6	3.1 ± 0.03
1	356 ± 4	219 ± 2	44 ± 0.4	2.9 ± 0.03
10	560 ± 8	254 ± 3	34 ± 0.3	2.8 ± 0.03

**Table 4:** Impedance (kΩ) measurements made with three sets of fixed resistors at different test frequencies.

Frequency/Hz	Value of Resistance/kΩ	Time/s											
		10	20	30	40	50	60	70	80	90	100	110	120
10	100	100.0	101.0	101.2	101.1	100.4	98.9	99.2	100.9	99.7	101.0	101.2	101.1
	500	501.3	502.3	505.2	501.4	504.8	500.1	494.5	501.3	495.3	500.7	500.2	501.2
	1000	1015.1	1001.8	1005.4	1005.8	1007.2	1002.7	1000.51	1002.0	1002.6	994.1	987.8	1000.1
60	100	100.1	100.3	101.0	100.0	99.3	98.9	98.9	98.8	99.0	98.9	100.2	100.7
	500	501.8	501.3	505.7	504.2	500.1	500.0	502.9	505.6	504.2	503.1	500.0	501.2
	1000	1010.0	1004.6	1003.1	1012.0	1005.4	1011.9	1005.4	1009.3	1001.4	987.9	988.7	1000.1
100	100	100.1	101.0	100.7	100.5	101.0	99.6	99.3	99.4	99.4	101.0	100.0	100.2
	500	501.1	500.3	505.7	500.1	501.2	501.8	502.3	501.0	500.1	500.7	500.0	501.1
	1000	1009.1	1007.9	1010.2	1011.8	1007.7	1010.1	1000.2	1012.0	1001.2	988.9	996.1	1000.0

**Table 5:** Comparison of TRSV detection methods.

Methods	Detection Time
Colloidal gold paper combined with RT-PCR	10 min
Real-time fluorescent PCR	0.5~2 h
SN-RT-PCR	3 h
RT-LAMP	1.5 h
Microfluidic impedance sensor	2 s

## Data Availability

Data available on request due to privacy.
